# Sex-specific dispersal and evolutionary rescue in metapopulations infected by male killing endosymbionts

**DOI:** 10.1186/1471-2148-9-16

**Published:** 2009-01-16

**Authors:** Dries Bonte, Thomas Hovestadt, Hans-Joachim Poethke

**Affiliations:** 1 University of Würzburg, Field Station Fabrikschleichach, Glashuettenstrasse 5, D-96181 Rauhenebrach, Germany; 2 Terrestrial Ecology Unit, Department of Biology, Ghent University, K.L. Ledeganckstraat 35, BE-9000 Ghent, Belgium

## Abstract

**Background:**

Male killing endosymbionts manipulate their arthropod host reproduction by only allowing female embryos to develop into infected females and killing all male offspring. Because the resulting change in sex ratio is expected to affect the evolution of sex-specific dispersal, we investigated under which environmental conditions strong sex-biased dispersal would emerge, and how this would affect host and endosymbiont metapopulation persistence.

**Results:**

We simulated host-endosymbiont metapopulation dynamics in an individual-based model, in which dispersal rates are allowed to evolve independently for the two sexes. Prominent male-biased dispersal emerges under conditions of low environmental stochasticity and high dispersal mortality. By applying a reshuffling algorithm, we show that kin-competition is a major driver of this evolutionary pattern because of the high within-population relatedness of males compared to those of females. Moreover, the evolution of sex-specific dispersal rescues metapopulations from extinction by (i) reducing endosymbiont fixation rates and (ii) by enhancing the extinction of endosymbionts within metapopulations that are characterized by low environmental stochasticity.

**Conclusion:**

Male killing endosymbionts induce the evolution of sex-specific dispersal, with prominent male-biased dispersal under conditions of low environmental stochasticity and high dispersal mortality. This male-biased dispersal emerges from stronger kin-competition in males compared to females and induces an evolutionary rescue mechanism.

## Background

Parasites can induce host population extinction through negative effects on population growth [1]. This effect can be amplified by the induction of host behaviour that stimulates parasite spreading [1-3]. Some bacterial endosymbionts that that are predominantly vertically transmitted from females to their offspring can be regarded as such parasites. They are widespread in arthropods and manipulate reproduction of their host [4,5]. The induced reproductive manipulations comprise parthenogenesis (i.e. infected virgin females produce daughters), feminization (infected genetic males reproduce as females), cytoplasmatic incompatibility (CI; in its simplest form the mating between infected males and uninfected females leads to the death of embryos), and male killing (i.e. infected male embryos die while infected female embryos develop into infected females). Male-killing imposes substantial costs at the individual level (both on infected females and on the males that mate with them) since the death of male offspring halves the number of viable offspring. Such male-killing endosymbionts are known from butterflies, ladybird beetles and flies, in which they affect sex ratio and related life-history parameters [5]. If both, endosymbiont transmission and host manipulation occur with near-perfect efficiency, infections can approach (near) fixation and host extinction will occur when all males are eliminated out of the population [6,7]. However, Randerson and colleagues [8] showed that hosts can avoid extinction by adaptive alterations of sexual behaviour, thereby raising their inclusive fitness. A prominent example comprises male-killing bacteria that trigger increasing male fatigue and female promiscuity in infected population of a butterfly [9].

Male killing endosymbionts clearly impair individual fitness of infected males (and implicitly of the males mating with them). Yet, the invasion of male-killers in a metapopulation has strong and more complex effects on the population structure. First, effective population sizes are strongly reduced due to skewed sex ratios [10]. This is expected to lead to a reduction in genetic variation [11], which subsequently reduces the effectiveness of selection against deleterious mutations [12] and the rate of adaptive evolution [13,14]. Secondly, endosymbionts reduce population density and thus relax intraspecific competition [15]. This relaxation is mainly among kin when eggs are laid in clutches and female offspring benefit from the death of their brothers by e.g. lowered resource competition in a later life phase [15,16]. Alternatively, male death enhances the fitness of their infected female siblings by prevention of inbreeding [17]. However, when competition takes place among all patch inhabitants male-killing endosymbionts reduce competition among offspring, thereby potentially benefiting surviving individuals within the local population indifferently of their infection status. This can lead, under certain conditions, to the spread of male-killing endosymbionts in metapopulations (by beneficial group trait selection) in absence of any explicit fitness compensation [18].

At any rate, male-killing endosymbionts have the ability to alter the demographic properties of their host population by inducing female-biased sex ratios. In combination with environmental factors related to dispersal mortality and demographic stochasticity these changes in population structure are expected to have a strong influence on the evolution of dispersal in spatially structured populations [19-25]. In a previous contribution [18], we already showed that the invasion of male killers selects for increased dispersal under conditions of low environmental stochasticity and high dispersal mortality. These evolved dispersal rates subsequently provoked extinction-colonization dynamics among patches in the metapopulation, thereby leading to stable infection frequencies (from here-on referred to as infection rates), metapopulation extinction of host and endosymbiont, or endosymbiont extinction only. Yet, because male-killing endosymbionts induce pronounced sex-specific effects on fitness and kin structure, they should also influence the evolution of dispersal differently in males and females. This is in agreement with the general predictions of Leturque & Rousset [26] that evolved changes in sex ratio may lead to higher dispersal rates and trigger the evolution of sex-specific dispersal. A theoretical background for the evolution of sex-biased dispersal is, however, poorly developed. Perrin & Mazalov [27] showed that mating systems are expected to be an important driver of sex-biased dispersal because sex-specific differences in potential reproductive success affect the balance between local resource mate competition and local resource competition.

When populations occupy spatially structured habitat, evolutionary changes in dispersal may rescue populations from (human induced) changes in habitat availability and quality [28]. These adaptive responses can indeed occur in fairly short time spans, as, for example, shown for wind dispersing arthropods [29,30] and vascular plants [31]. Metapopulation curing, i.e. the deterministic extinction of parasites but not the host, under environmental conditions that select against dispersal [18] could be another prominent example of such an evolutionary rescue. Because male killing endosymbionts generate strong bias in sex ratio, and increase dispersal rates considerably [18], we here explore the evolutionary mechanisms leading to male-biased dispersal in infected populations. Secondly, we show that a male-bias in dispersal is responsible for higher rates of metapopulation curing (host extinction) compared to populations with sex-indifferent dispersal.

## Results

We build an individual based simulation model that allowed the evolution of sex-specific dispersal strategies in a metapopulation consisting of 100 patches with carrying capacity *K*, inhabited by sexually reproducing, polygynous organisms. Dispersal is accompanied by costs (*μ*) that relate to patch isolation. Environmental stochasticity is modelled by a standard deviation (*σ*) around the number of offspring (*λ*). Population dynamics follow logistic growth, endosymbionts are maternally transmitted from mother to daughters (male offspring die during the embryonic stage when the mother is infected). Dispersal strategies were determined by two sex-specific dispersal alleles.

**Figure 1:**
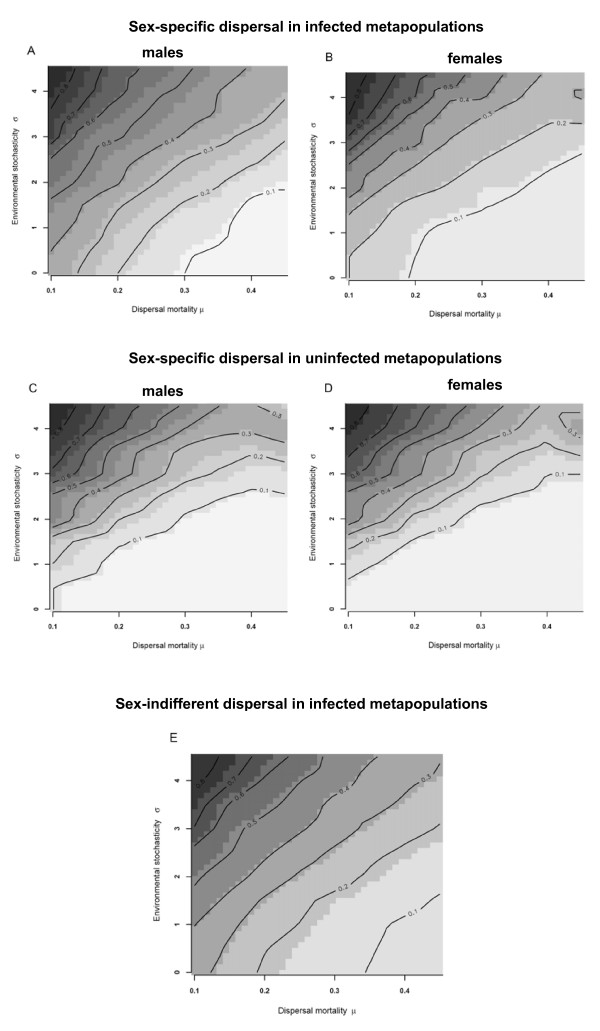
**Sex specific dispersal rates**. Dispersal probabilities for sex-specific strategies in infected (A males; B females) and uninfected metapopulations (C males; D females) and sex-indifferent strategies in an infected metapopulation (E). The x-axis gives dispersal mortality (*μ*), the y-axis environmental variability (*σ*).

Mean dispersal probabilities reached equilibrium after less than 2000 generations. Similarly, sex ratio and the proportion of infected individuals stabilised after this number of generations. In general, our simulation results confirmed the evolution towards higher dispersal probabilities under higher environmental stochasticity (*σ*) and lower costs of dispersal (*μ*) (Fig 1). This pattern holds for males (Fig 1A) as well as females (Fig 1B), but a considerable male-bias in dispersal rate was observed (Fig 1A*versus *1B; Table 1). Similar patterns were found for simulation experiments with uninfected metapopulations, although overall dispersal probabilities as well as male-bias are considerably lower in the latter (Fig 1C, 1D). When simulations were run for sex-indifferent dispersal strategies (i.e., one allele coding for dispersal propensity in males and females) in infected metapopulations (Fig 1E) we noticed the evolution of increased dispersal probabilities, particularly under low environmental stochasticity and high dispersal costs.

**Table 1:** Bias in sex-specific dispersal

**Scenario**	**Bias**
no infection, no reshuffling	0.55 ± 0.04
no infection, reshuffling	0.55 ± 0.04
infection, no reshuffling	0.61 ± 0.06
infection, reshuffling	0.51 ± 0.04

Bias (male dispersers/total number of dispersers) in sex-specific dispersal for the different investigated scenario (mean and SD over the considered ranges of *σ *(0 ≤ *σ *≤ 4.5) and *μ*(0.1 ≤ *μ *≤ 0.45)).

**Figure 2:**
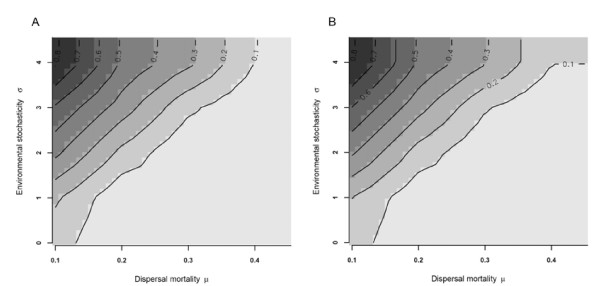
**Sex specific dispersal rates after eliminating kin competition**. Dispersal probabilities for sex-specific strategies (A males; B Females) after reshuffling (elimination of kin competition). The x-axis gives dispersal mortality (*μ*), the y-axis environmental variability (*σ*).

In order to eliminate effects of kin competition, but retaining all other characteristics of local populations, we performed a reshuffling experiment. By this, individuals within each patch were replaced by individuals of the same sex and with the same infection status randomly selected from the entire pool of individuals in the metapopulation. The genetic structure of the metapopulation is consequently homogenized and kin-competition eliminated. This leads to a decrease in dispersal probability in all scenarios. The decline was especially pronounced under conditions of high dispersal mortality and low environmental stochasticity (compare Fig 1A, B with Fig 2A, B). More interestingly, reshuffling removed the male bias of dispersal (Table 1) and dispersal patterns became similar for both sexes (Fig 2A*versus *Fig 2B).

**Figure 3:**
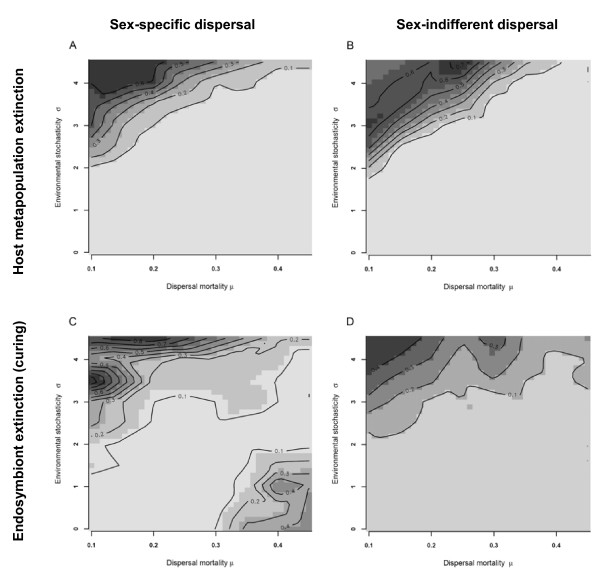
**Metapopulation extinction rates**. Metapopulation (upper panels A, B) and endosymbiont extinction (lower panels C, D) probabilities for sex-specific (left panels, A, C) and sex-indifferent strategies (right panels B, D). Note decreased metapopulation extinction and increased curing under scenarios with evolution of sex-specific dispersal. The x-axis gives dispersal mortality (*μ*), the y-axis environmental variability (*σ*).

Sex-indifferent dispersal strategies had a minor rescue-effect on the entire metapopulation extinction probability, with a slight shift towards decreasing extinction probabilities under conditions that select for high dispersal (Fig 3A, B). In contrast, allowing the evolution of sex-specific dispersal strategies increases the chance of endosymbiont extinction while the host metapopulation survives. As evident from Fig 3C and Fig 3D, the evolution of male-biased dispersal induced curing (only endosymbiont and not host extinction) especially under high dispersal mortality and low environmental stochasticity but also increased curing under conditions of high environmental stochasticity.

## Discussion

Strong environmental fluctuations and low dispersal mortality are well-acknowledged factors that support the evolution of high emigration probability [19-25,32,33]. Our simulations suggest that the presence of male-killing microbial infections should increase overall dispersal rates, too. When dispersal is assumed to be independent of sex, dispersal probabilities showed a significant increase, particularly for those scenarios otherwise favouring low dispersal (i.e. high dispersal mortality and low environmental stochasticity). However, if allowed for sex-specific dispersal, endosymbionts induced pronounced male-biased dispersal rates. Effects of male-killing endosymbionts on demography and life history in e.g. tropical butterflies [34], flies [35] and ladybird beetles [36] are documented. No attempts have been made, so far, to link infections by male-killing endosymbionts to spatial population structure and dispersal. Our results suggest that this would be a worthwhile endeavour.

The higher rates of patch extinction induced by male-killing endosymbionts compared to metapopulations of uninfected hosts [18], as well as the benefits of relieved competition in patches that are founded shortly after patch extinction select for higher dispersal rates [24,37-39]. The relationship between extinction rate and dispersal might, however, become hump-shaped with very high extinction rates selecting for reduced dispersal [40]. Yet in our simulations, as well as in simulations without infection [24], local extinction rates so high as to select for reduced dispersal never emerged because the system rapidly went globally extinct under such conditions.

Sex-biased dispersal is documented to originate from strategies related to inbreeding avoidance, inbreeding depression, asymmetrical mating systems, social structure, or sex-specific dispersal costs [27,33,41]. More generally, the small effective population size and the tighter kin-structure in infected metapopulations due to the rarity of males explain the evolution towards overall higher dispersal rates, compared to metapopulations without male-killing endosymbionts. This particularly holds under conditions of high dispersal mortality and low environmental stochasticity. However, theory generally predicts that strong kin-competition selects against a sex-bias in dispersal [41], while in our simulations we demonstrate that strong kin-competition is responsible for the emergence of male-biased dispersal. The explanation for this apparent contradiction can be found in the unbalanced cost-benefits for dispersal in males and females. In Gandon's system [41], males and females played the same game, i.e. they both competed for space and mates and have the same costs of dispersal. Taylor [42] showed, however, that deviations from these conditions, i.e. sex-specific costs of dispersal and different relatednesses for the two sexes, induce sex-specific dispersal. The latter assumed that a sex bias in relatedness should only be important in haplodiploid populations, but not in diploid organisms. In our system, however, male-killing endosymbionts strongly affect within-population relatedness, because under high infection rates the (few) males in a patch have higher probabilities to share mothers than females. This is due to the fact that in populations with an infection rate *I *only a fraction of 1-*I *females produce sons, while all females produce daughters. Like for Taylor's haplodiploid system, the most related sex (females in a haplodiploid system, but males in our case) should thus evolve higher dispersal rates than patchmates from the other sex.

One could also speculate that dispersing males take a greater risk of mating with infected females as the very existence of males in a patch may indicate reduced infection rates in that patch. However, consequences of such an increase in the cost of dispersal could not be observed. Probabilities of mating with uninfected females were not higher for males in their natal patches, because populations may persist at high infection frequencies [18]. Moreover, the particularly strong kin-competition for males overrules this potential (and sporadic) increased chance of finding uninfected females.

Leturque & Rousset [26] showed that sex-specific dispersal rates may evolve when reproductive values vary among genotypes and when relatedness is high. Interestingly, their model also shows that the incorporation of sex-biased dispersal may lead to a biased sex ratio in offspring production in finite populations and in populations experiencing spatial heterogeneity in habitat quality. In our model, changes in the sex ratio are due to male-killing endosymbionts and lead to higher and sex-biased dispersal. Yet, we could expect that the changes in sex ratio might trigger an evolutionary response also in offspring sex ratio. If a female can recognize its own infection status, infected females should clearly shift the sex ratio in favour of female offspring as the male offspring is killed anyways. Uninfected females should in contrast produce more male offspring as they would have more fitness contribution via sons. However, that only holds for infected populations because in uninfected ones the sex ratio is 1:1. So ideally, such females should response to both, their own infection status and the infection status of the population.

In well connected metapopulations with high inter-patch dispersal, metapopulation extinction probabilities are hardly affected by dispersal. Under these conditions the high dispersal leads to high recolonization rates of empty patches but also to a rapid spread of infections into uninfected populations [18]. Consequently, endosymbiont extinction probabilities are only slightly affected by sex-specific dispersal when dispersal is generally high (even in females). By contrast, deterministic curing of metapopulations increases with sex-specific dispersal strategies. The evolution of sex-specific dispersal leads to considerably lower dispersal probabilities of females (compared to simulations with sex indifferent dispersal) under environmental conditions characterized by high dispersal mortality and low environmental stochasticity. This leads to a comparable decline in recolonization rates of patches by infected females and the spread of infections. The evolution towards male-biased dispersal in infected metapopulations – promoted by the benefit of reduced kin-competition in males – can consequently be regarded as an evolutionary rescue as it increases the probability of curing for the entire metapopulation.

In our simulations we assume global dispersal as opposed to e.g. nearest neighbour dispersal, reflecting airborne dispersal in e.g. arachnids and insects. Because kin-competition is the dominant driver behind the evolution of sex-specific dispersal, we could expect limited dispersal distance (i.e., nearest neighbour dispersal), which maintains some kin-competition even after dispersal, to have a strong influence on the evolutionary dynamics. However, simulation experiments for uninfected metapopulations [23] as well as our own simulations (Bonte D, Hovestadt T, Poethke HJ, unpub. data) show that the choice of dispersal-mode has little influence on the evolution of ES dispersal probabilities. This is due to intrinsically high dispersal rates and the absence of any spatial autocorrelation in environmental stochasticity. This demonstrates that kin-competition is predominantly generated through changes in local population structure, i.e. the (much) reduced effective population size in infected populations and not by limited dispersal distance.

Kin-competition is documented to select for increased dispersal rates when dispersal cost is high and/or when spatio-temporal variability of environmental conditions is low [21,25,42-45]. Male-killing endosymbionts induce strong kin-competition under these environmental conditions, which lead to strong sex-biased dispersal in infected metapopulations. Paradoxically, their induced negative feedbacks on female dispersal rates eventually decrease their own persistence in a host metapopulation. Adaptive dispersal or genotype-biased dispersal strategies are already known to rescue metapopulations from extinction [46-48]. Here we show that evolution of sex-specific dispersal could enhance persistence of hosts that experience infection by male-killing endosymbionts in two different ways: (i) by decreasing host extinction probabilities (minor effect) and (ii) by inducing curing of the host (major effect) through the combined action of increased male and decreased female dispersal. In addition to the finding that adaptive dispersal can promote the evolution of parasite resistance [49], we here show that an evolutionary response of dispersal strategies to male-killing endosymbiont infection may already as such be an adaptation to escape male-killing endosymbiont infections.

## Conclusion

Male killing endosymbionts induce the evolution of sex-specific dispersal, with prominent male-biased dispersal under conditions of low environmental stochasticity and high dispersal mortality. This male-biased dispersal emerges from kin-competition, which is (much) stronger in males because they are all offspring of the (few) females of their high relatedness. In addition, the evolution of sex-specific dispersal rates induces an evolutionary rescue mechanism by either decreasing endosymbiont fixation probabilities (which subsequently would lead to the crash of the host metapopulation) under conditions of high environmental stochasticity, or increasing endosymbiont extinction (curing) under conditions of low environmental stochasticity.

## Methods

### The model

#### The landscape

For our simulation experiments we use an extended version of an individual-based model [23-25] of insect dispersal in patchy landscapes of *n *(= 100) habitat patches with equal carrying capacities *K *(= 100).

#### The individual

Each individual is characterized by its sex, its affiliation with a specific patch (*i*), and by four alleles at two different diploid loci that determine male (*d*_*m*_), respectively female (*d*_*f*_) dispersal propensity (see below). At initialization allele values are drawn randomly from a uniform distribution [0–1]. Further, individuals are characterized by their infection status (infected versus uninfected), which they solely inherit from their mother.

#### Population dynamics

Local population dynamics are governed by density-dependent reproduction of individuals. After mating with a randomly drawn local male (thus assuming polygyny), a female gives birth to Λ offspring, where Λ is a Poisson-distributed number with a patch- and time-specific mean, Λ_*mean*_*(t*, patch). For each generation, the mean value of Λ_*mean*_*(t*, patch) is drawn from a lognormal distribution with mean *λ *and a standard deviation *σ *(0 ≤ *σ *≤ 5). In our simulations, *λ *was set to 4, a value typical for arthropod demography [50]. *σ *subsequently determines the degree of environmental fluctuations which are assumed to be uncorrelated in space and time. Offspring are randomly assigned to the male or female sex, but male offspring die immediately after conception if the mother is infected. Remaining offspring develop into mature individuals with a density-dependent survival probability *s*:

(1) $$s=\frac{1}{\left(1+aN_{i}\right)}$$

with $\text{with }a=\frac{\lambda -1}{K}$

Here *N*_*i *_represents the population size in patch *i*. *K is *the carrying capacity of each patch in the metapopulation. It is important to recognize that the daughters of an infected mother do not exclusively benefit from the death of their brothers. Yet, in groups with infected females, population growth increases as female offspring are released from competition by males.

#### Dispersal

In our model, individuals simultaneously disperse before mating and production of offspring; each individual has only one opportunity to disperse. Dispersing individuals die with a probability *μ *(dispersal mortality), regardless of patch origin. For each individual its emigration probability *d *is determined by the mean value of their two sex-specific dispersal alleles, (*d*_*m*,1_+*d*_*m*,2_)/2 respectively (*d*_*f*,1_+*d*_*f*,2_)/2. We assume global dispersal; that is, a successful disperser reaches a patch in the landscape (except its home patch) with the same probability (1-*μ)*/(*n-1*). Dispersal probability is thus unconditional, i.e. we assume that dispersal decisions are not based on patch condition (e.g. infection rate, density or sex ratio). However, dispersal alleles were allowed to change by mutation, thus allowing for the evolution of sex-specific dispersal strategies To promote greater variability of genotypes in the first generations and to reduce the influence of mutations on the stability of the final result, we let mutation rates exponentially decrease from ~0.1 to <0.001 over the course of the simulation experiments (5000 generations; see e.g. [24]). A mutation comprises a change into a new random value from the uniform [0–1] distribution. For the case of symmetric, i.e sex-indifferent dispersal we assume that dispersal is governed by one locus only determining male as well as female dispersal propensity.

#### Simulation experiments

To infer how the presence of infections in the metapopulation influenced the evolution of dispersal probability in male and female hosts, we compared results of simulation experiments with and without endosymbiont infection. Experiments with infections were run with sex-specific as well as with symmetric dispersal behaviour. For both sets of experiments we estimated the metapopulation extinction probability (host and endosymbiont go both extinct) and the probability of endosymbiont extinction only ('curing'). Simulations were run with an initial fraction of infected females of *I *= 0.10 randomly distributed over patches. Additional simulations showed that the initial infection rate did not influence trait evolution (Bonte D, unpublished data). Because (i) we only observed an effect of carrying capacity on metapopulation dynamics and trait evolution when *K *<< 100; and (ii) typical insect habitats can be expected to rarely have capacities below K = 100 [51], we ran simulations only for values of *K *= 100.

Simulations were run for different combinations of dispersal mortality (*μ *= 0.1, 0.15....0.45; 8 values) and environmental stochasticity (*σ *= 0, 0.5, 1, 1.5 ...4.5; 10 values) resulting in a total of 80 scenarios for each of the simulation experiments described above. All scenarios were replicated 100 times. Global host as well as endosymbiont extinction probability was calculated as the number of simulation runs with metapopulation extinction divided by the total number of replicates for each scenario. Equally, the probability of curing was estimated by dividing the number of cured populations by the number of surviving host populations. In order to test the influence of kin-competition on the observed patterns in dispersal evolution, infection rate, and population dynamics, we applied a reshuffling algorithm by which local kin-structure is destroyed [24]. Here, individuals within each patch *i *were replaced by individuals of the same sex and with the same infection status randomly selected from the entire pool of individuals in the metapopulation. This experiment was also performed for the 80 scenarios described above with 100 replicates each.

#### Model restrictions

In our simulations, we do not allow for the evolution of host resistance. As shown by Dyer & Jaenike [52], endosymbionts may have only limited capacity to counter newly evolved host resistance because of their small effective population sizes [53]. Yet the most influential restrictions are probably the applied mating system and the dispersal strategy. Because endosymbionts infections are predominantly associated with arthropods, we assume polygyny since this is the most common mating system in insects and other arthropods [54]; our simulation results would certainly be very different for a monogamous mating system. Despite evidence that male-killing bacteria may affect sexual selection [9,55], we only allowed single mating events in order to provide straight mechanisms of inheritance.

## Authors' contributions

The work presented here was carried out in collaboration between all authors. DB conceptualized the research questions, implemented the model, analyzed the data, interpreted the results and wrote the paper. TH and HJP designed an earlier version of the model, discussed analyses, interpretation, and presentation. All authors have contributed to, seen and approved the manuscript.
